# Measuring liver fat fraction with complex-based chemical shift MRI: the effect of simplified sampling protocols on accuracy

**DOI:** 10.1186/s12880-019-0311-y

**Published:** 2019-02-08

**Authors:** Alexander J. Procter, Julia Y. Sun, Paul N. Malcolm, Andoni P. Toms

**Affiliations:** Norfolk and Norwich University Hospital NHS Foundation Trust, Colney Ln, Norwich, NR4 7UY UK

**Keywords:** ROI, Liver, PDFF

## Abstract

**Background:**

The assessment of liver percentage fat fraction (%FF) using proton density fat fraction sequences is becoming increasingly accessible. Previous studies have tended to use multiple small ROIs that focus on Couinaud segments. In an effort to simplify day-to-day analysis, this study assesses the impact of using larger, elliptical ROIs focused on a single hepatic lobe. Additionally, we assess the impact of sampling fewer transhepatic slices when measuring %FF.

**Methods:**

Retrospective analysis of prospectively obtained images from 34 volunteers using an IDEAL IQ sequence. Two observers independently measured %FF using three different protocols: freehand whole-liver ROI (fh-ROI), elliptical-ROI on the right lobe (rt-ROI) and elliptical-ROI on the left lobe (lt-ROI).

**Results:**

Inter-observer reliability for all measurements techniques was ‘excellent’ (Spearman’s rank correlation coefficients 0.81–0.98). There was a significant difference (Paired Wilcoxon Test: *p* < 0.001) between the median %FF obtained using fh-ROI when compared to the rt-ROI method, the maximum mean difference between the two techniques was 2.79% (95% CI). For all sampling methods a Kruskall-Wallis analysis demonstrated no significant difference in mean %FF when the number of slices sampled was reduced from 11 to 1. The mean coefficient of variance increased when more slices were sampled (3 slices = 0.1, 11 slices = 0.17, *p* < 0.001).

**Conclusion:**

Simplified ROIs focused on one hepatic lobe provide %FF measurements that are unlikely to be sufficiently accurate for use in clinical practice. Freehand whole-liver ROIs should be used in preference.

A single freehand ROI measurement taken at the level of the hepatic hilum yields a %FF that is representative of the mean whole liver % FF. Multiple slices are needed to measure heterogeneity.

## Background

Non-alcoholic fatty liver disease (NAFLD) is the most common chronic liver disease in developed nations with a prevalence of 20–30% in adults [1] and as much as 70–91% in high-risk patients such as those with obesity [2] and diabetes [3–5]. Among patients who have evidence of hepatic steatosis a proportion will go on to develop non-alcoholic steatohepatitis (NASH) and a subpopulation will progress [6] to cirrhosis, end-stage liver disease or related complications such as hepatocellular carcinoma (HCC) [7, 8].

The current gold standard for diagnosing and grading hepatic steatosis is core biopsy. This method is invasive and prone to sampling errors caused by heterogeneity of liver fat deposition [9]. Alternative techniques that have been developed for the assessment of liver percentage fat fraction (%FF) include MRI and MR spectroscopy (MRS). These are non-invasive and thus more suitable for longitudinal follow-up as well as allowing larger regions of interest (ROI) to be sampled thus reducing sampling error [9].

A number of MRI-based methods currently used to quantify liver %FF have been validated by comparison to histological sampling and MRS [10–12]. MRS is regarded as the most accurate MRI method for the measurement of liver %FF. [9] MRI techniques (e.g. dual-echo Dixon or the more recent complex-based approaches that enable measurement of proton density fat fraction (PDFF)) have been shown to provide reliable quantification of liver %FF and are more widely available than MRS [13–17].

One widely used PDFF technique is the water–fat separation method “iterative decomposition of water and fat with echo asymmetry and least squares estimation” (IDEAL) [18]. Co-registration and recombination of fat and water images provides pixel by pixel fat fraction maps [19]. Measurement of fat fraction requires manual selection of an ROI on one or more slices, providing one or more values of average liver %FF. The sample of liver examined will depend on the area of the ROIs chosen and the number of slices of liver interrogated. Most of the previously described methods have used ROIs that are very small relative to the size of the liver [20] or focus on using ROIs centred on each Couinaud segment [21].

Ideally a method that samples all of the imaged liver parenchyma should be used, indeed some semi-automated computer-based post-processing methods that do this have been described [20]. However, these tools use image recognition software that is not widely available and often still requires manual corrections. For day-to-day analysis in a non-research setting post-processing commonly involves the manual drawing of an ROI using software provided by the MRI manufacturer. In this situation if the entire liver parenchyma were to be sampled this would require individual freehand ROI to be drawn around the liver on every slice where parenchyma can be seen, but this is time-consuming and labour-intensive.

Evaluation times could be reduced by employing the following techniques:

- Using single large elliptical ROIs that approximate to the size of a hepatic lobe, rather than laboriously drawing a freehand ROI around the liver edge.
- Reducing the number of slices sampled, rather than aggregating ROIs which have been drawn on every slice that contains liver parenchyma.

However, in theory these simplified protocols increase the chance of sampling error.

The purpose of this study is to assess the accuracy of %FF measurements obtained when using these simplified sampling protocols (i.e. large elliptical ROIs and reducing the number of slices sampled). Freehand drawn ROIs that sample the whole liver will be used as a reference standard.

## Methods and materials

Ethical approval for this study was obtained from the local Research Ethics Committee. Informed written consent was obtained from each participant. 34 volunteers were recruited from Radiology department staff at the Norfolk and Norwich University Hospital. An equal number of male and female volunteers were selected from a variety of different body mass indices (BMIs), these ranged from normal (BMI 18–25) to obese (BMI > 30). The population used in this study was originally recruited to take part in two test-retest reliability studies investigating whole body muscle and body-compartment fat quantification [22, 23].

### Magnetic resonance imaging

Each participant underwent MR imaging of the liver on a MR750w wide bore 3 T MR machine (General Electric Medical Systems Ltd., Hatfield, UK) using an integrated quadrature body coil. IDEAL IQ (3D technique, NEX: 0.5, Bandwidth: 111.11 kHz, TEs per scan: 6, Number Shots: 2, Minimum TE: 0.9 ms, Maximum TE: 4.6 ms, Auto Flip angle: 3°, Echo train length: 3, TR: 6 ms, FOV: 50.0 × 50.0 cm, Slice Thickness: 10.0 mm) MR images were acquired with the volunteers supine and with arms by their sides. Fourteen axial slices were taken through the region of the liver and used for our analysis.

### Regions of interest (ROI)

**Fig. 1:**
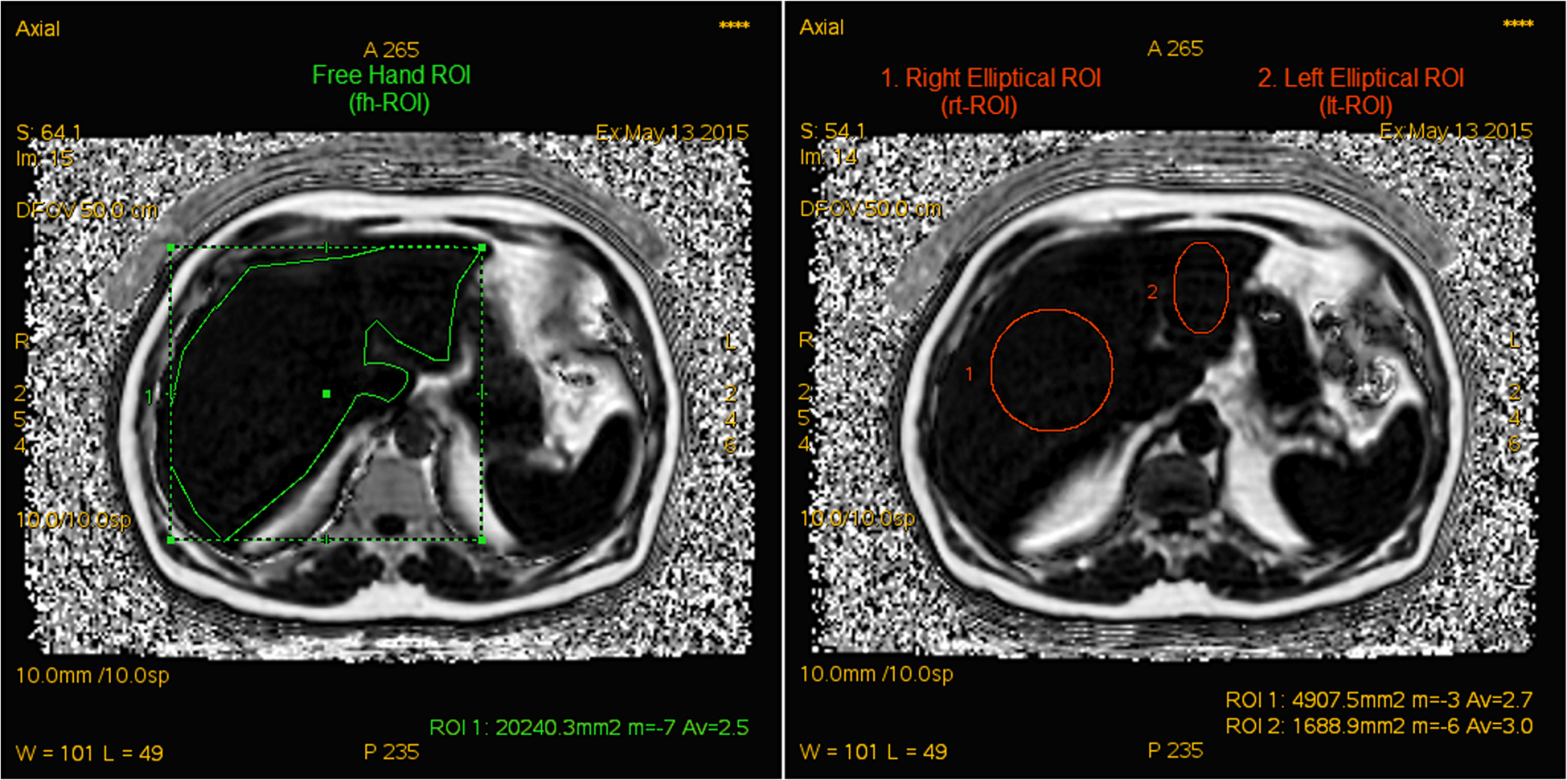
Screenshots illustrating examples of the different region of interest (ROI) placements for the freehand whole liver, the elliptical right lobe and the elliptical left lobe. The elliptical ROIs were individually drawn on each slice available and aimed to contain as much liver parenchyma as possible

Two observers (radiology residents) independently measured the %FF using two different protocols on a dedicated post-processing workstation (AW VolumeShare 5, GE Healthcare). The first protocol was to generate a freehand whole liver ROI (fh-ROI) drawn around the margins of the entire liver excluding fat at the porta hepatis and falciform ligament. The second protocol was to draw an elliptical ROI over the centre of the right (rt-ROI) and left lobes (lt-ROI) of the liver respectively aiming to create as large an ellipse as possible whilst keeping it within the boundaries of each lobe (Fig. 1). The inferior vena cava and portal vein were excluded from the ROIs but smaller intrahepatic vessels and bile ducts were not excluded.

**Fig. 2:**
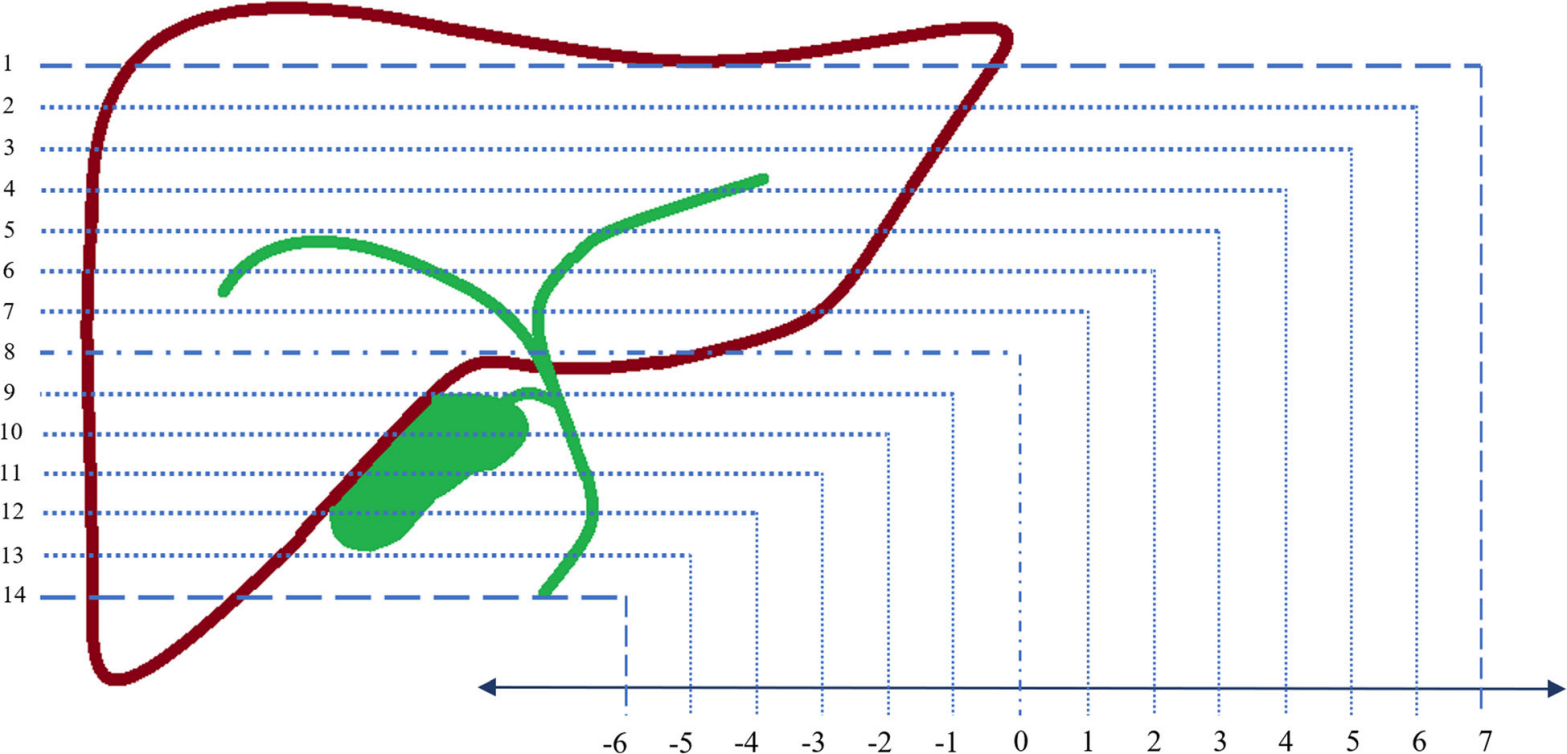
Schematic diagram demonstrating how the slices obtained from each acquisition were standardised according to the position of the hepatic hilum. The axial slice containing the hilum of the porta hepatis was set as “slice 0” with the more cranial slices recorded as positive and the more caudal slices as negative, positions relative to the hilum

The mean %FF within each type of ROI was recorded for each observer and measurements were made on all fourteen slices where there was identifiable liver parenchyma. To allow for the variable amount of liver covered by each individual scan, and to facilitate slice-to-slice comparison between individuals, a note was made of the axial slice number that included the hilum of the porta hepatis. For the purposes of further analysis this hilar slice was set as “slice 0” with the more cranial slices recorded as positive, and the more caudal slices as negative, positions relative to the hilum (Fig. 2). As a result of anatomical differences and some variations in liver coverage, this ‘hilar correction’ generated slice positions ranging from − 10 through to + 9.

### Statistical analysis

Statistics were performed using the R software environment [24]. Q-Q plots and Shapiro-Wilk tests for normality were performed. Descriptive statistics included a coefficient of variation (CV), which was calculated as a relative standard deviation of the fat fraction and presented as a percentage. Inter-observer reliability was measured using Spearman’s rank correlation coefficients and 95% limits of agreement derived from Bland-Altman plots. Kruskal-Wallis tests, with subsequent post-hoc analyses, were used for multiple hypothesis testing of non-parametric data.

## Results

**Fig. 3:**
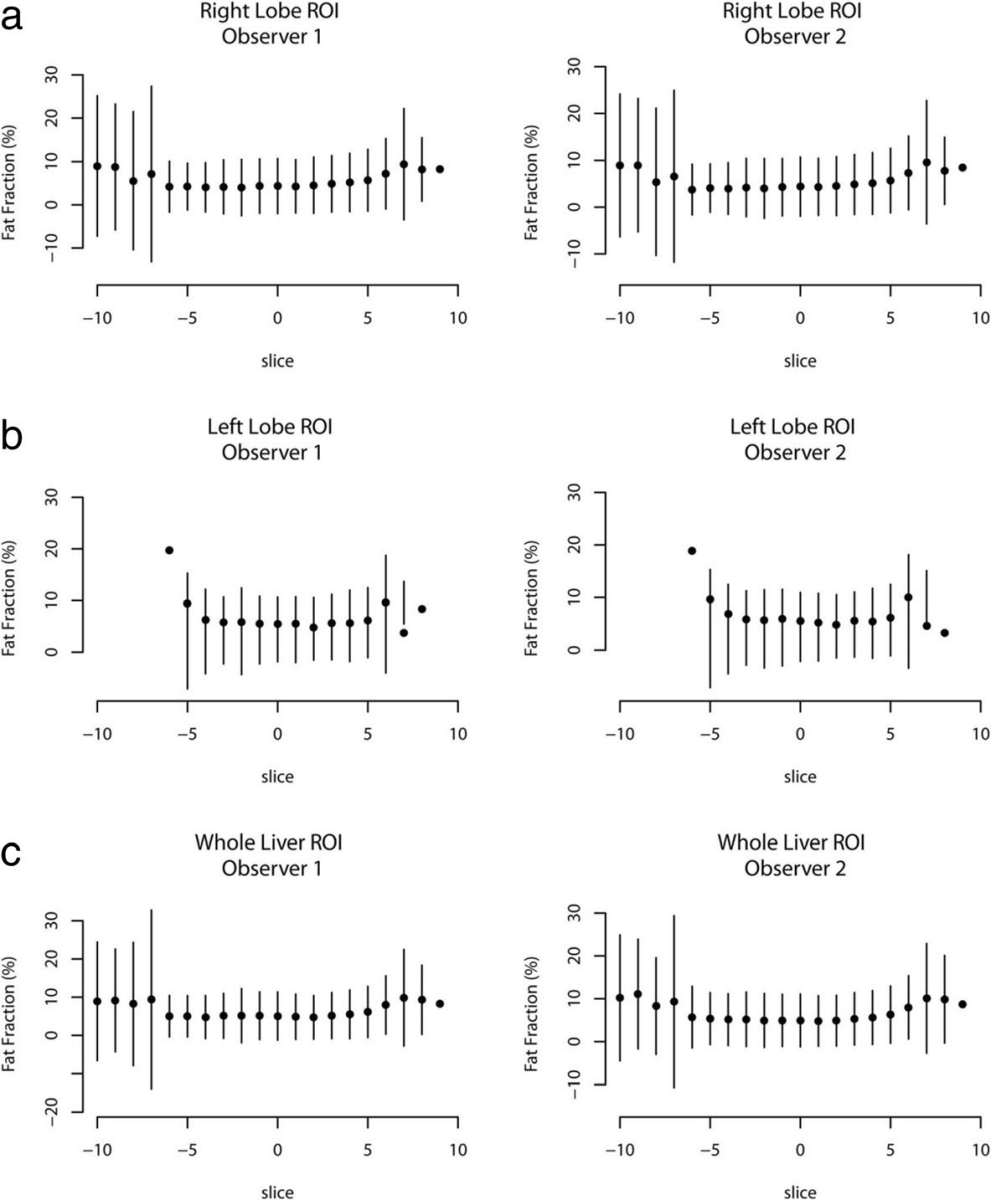
Graphs demonstrating the mean % fat fraction, and standard deviation, for each slice through the liver from most caudal (negative slice number) to most cranial (positive slice number). **a** demonstrates data from an elliptical region of interest (ROI) placed on the right lobe. **b** demonstrates data from an elliptical ROI placed on the left lobe. **c** demonstrates the data from freehand whole liver ROIs. Deviations in data points at the cranial-most and caudal-most slices may be caused by movement artefacts and partial volume effects

Slice-by-slice mean %FF and the standard deviation were calculated for the fh-ROI, rt-ROI and lt-ROI measurements obtained from all volunteers. When these calculations were plotted against slice number, deviations from the mean and increases in standard deviation were demonstrated at the most cranial and most caudal slices (Fig. 3).

**Fig. 4:**
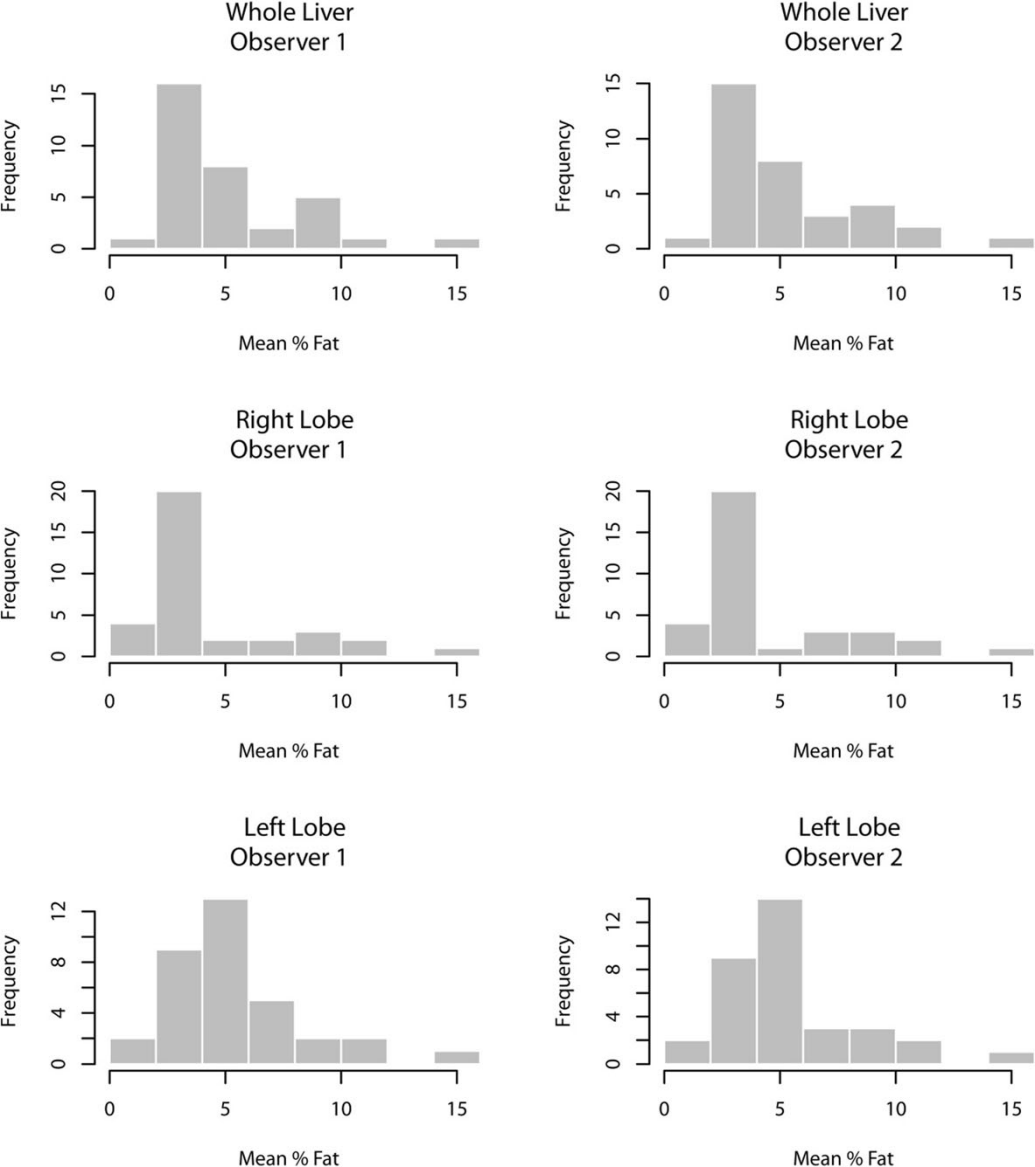
Frequency histograms of the three regions of interest (ROIs), drawn by each of two observers, demonstrating non-parametric distributions

Frequency histograms of the mean %FF demonstrated a skewed distribution for all sampling methods (Fig. 4). Q-Q plots and Shapiro-Wilk tests for normality confirmed that all samples failed to conform to a parametric distribution (Shapiro-Wilk *P* = 0.001 or less for all datasets at significance level of 0.05) and therefore non-parametric descriptive and hypothesis testing statistics were used.

The median %FF for fh-ROIs was 4.0 (IQR 3.1–6.0) for observer 1 and 4.51 (IQR 3.2–6.2) for observer 2. The medians for rt-ROI and lt-ROI were 3.31 (IQR 2.4–5.6) and 4.54 (IQR 3.9–6.5) respectively for observer 1, and 3.31 (IQR 2.4–5.6) and 4.75 (IQR 3.7–6.1) for observer 2. The Spearman’s rank correlation coefficients demonstrated “almost perfect” agreement [25] between the two observers with reasonably narrow 95% limits of agreement derived from the Bland-Altman plots (Table 1).

**Table 1:** Table summarising the descriptive statistics, mean differences, limits of agreement and Spearman’s rank correlation coefficient between observers

Sampling method	Observer	Median %FF (95% CI for median)	IQR	Mean Difference (95% limits of agreement)	Spearman’s rank correlation coefficient
Whole Liver	1	4.00 (3.4, 4.8)	3.1–6.0	−0.09 (− 0.9, 0.7)	*ρ* = 0.95 (*p* < 0.001)
	2	4.51 (3.2, 5.5)	3.2–6.2		
Elliptical ROI right lobe	1	3.31 (2.5, 3.7)	2.4–5.6	0.05 (−0.2, 0.3)	*ρ* = 0.98 (*p* < 0.001)
	2	3.31 (2.4, 3.6)	2.4–5.6		
Elliptical ROI Left lobe	1	4.54 (4.0, 5.6)	3.9–6.5	−0.03 (−0.8, 0.8)	*ρ* = 0.81 (*p* < 0.001)
	2	4.75 (4.0, 5.4)	3.7–6.1		

There were significant differences demonstrated between the three sampling techniques (Kruskal-Wallis test *P* = 0.032 for observer 1, and *P* = 0.036 for observer 2). Post hoc pairwise analysis with Wilcoxon Rank Sum tests performed with Holm correction for multiplicity in paired data demonstrated that the significant differences were between rt-ROI and fh-ROI, as well as between rt-ROI and lt-ROI (Table 2). The difference between the mean rt-ROI and fh-ROI values was found to be at most 2.79%.

**Table 2:** Table comparing freehand whole liver region of interest (ROI) and elliptical-ROIs for the right and left lobes performed by both observers demonstrated significant differences when tested with the Kruskal-Wallis test (*P* = 0.032 and 0.036)

Sampling method compared	Observer	Diff in median (IQR)	Mean difference (95% limits of agreement)	Coefficient of repeatability	Significance^a^
Whole vs right ellipse	1	0.50 (0.29–0.99)	0.62 -0.58, 1.82	1.20	< 0.001
	2	0.56 (0.26–1.1)	0.80 -0.60, 2.19	1.39	< 0.001
Whole vs left ellipse	1	0.72 (0.36–1.56)	-0.32 - 3.18, 2.54	2.86	0.44
	2	0.71 (0.45–1.5)	-0.22 - 2.96, 2.51	2.74	0.52
Right vs left ellipse	1	1.15 (0.75–1.86)	-0.94 -4.40, 2.52	3.46	0.007
	2	1.3 (0.60–1.89)	-1.01 -4.2, 2.17	3.19	0.001

^a^Post-hoc paired Wilcoxon Rank Sum Test with Holm correction

Post-hoc tests revealed these differences to lie between freehand whole liver ROIs and elliptical right lobe ROIs and between the elliptical ROIs of the right and left lobes

**Fig. 5:**
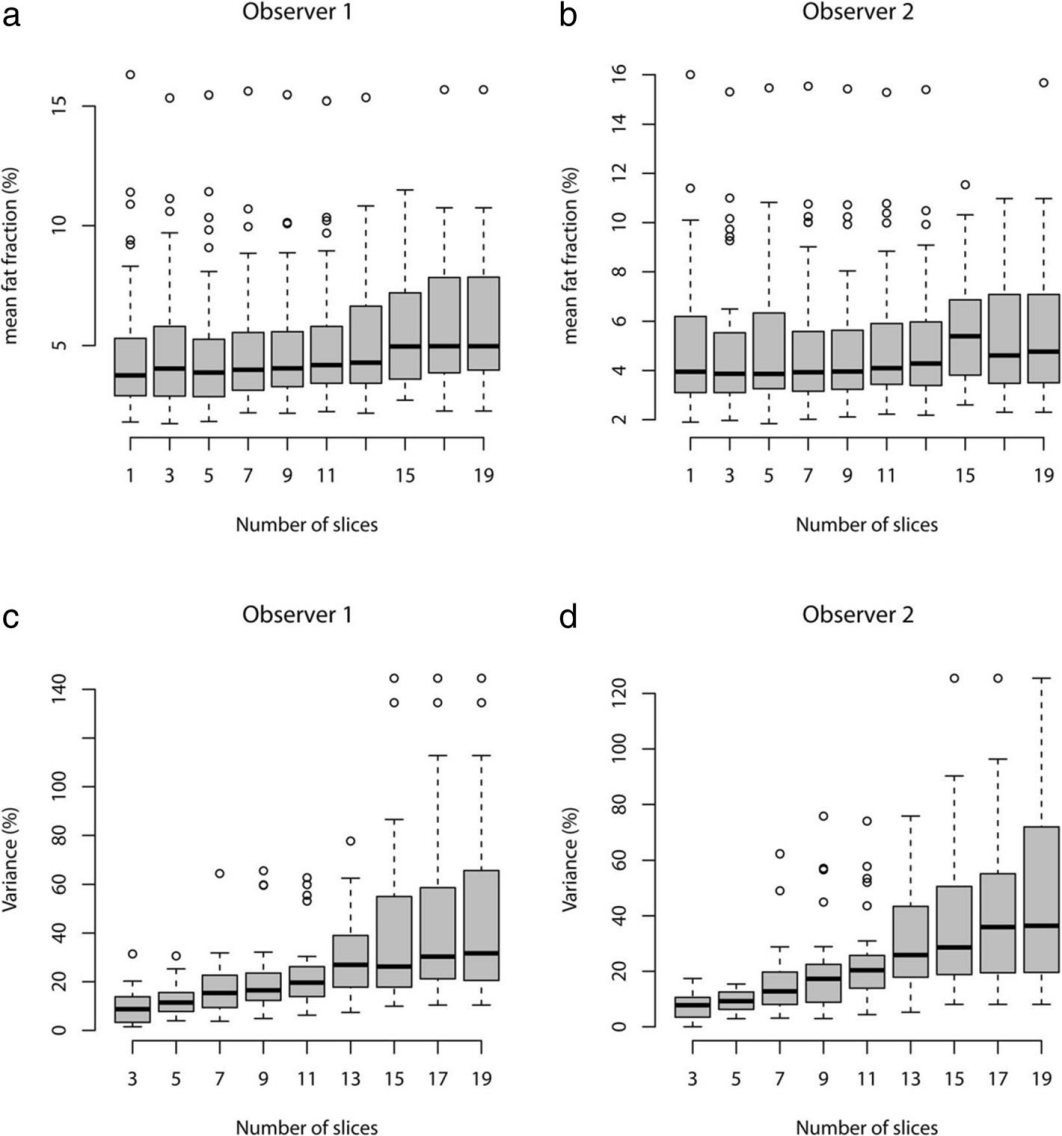
Box-plots comparing the mean percentage fat fraction (%FF) derived from datasets comprising progressively more slices either side of the hepatic hilum for the freehand whole liver regions of interest (ROIs) for observers 1 (**a**) and 2 (**b**) with equivalent plots for coefficients of variance (**c** & **d**). There was no significant difference in mean %FF (**a** & **b**) but there was a significant difference in coefficients of variance from one dataset to another (**c** & **d**)

The mean %FF and %FF variance were then compared for datasets comprising 1 to 19 slices in two slice increments. For each category interval one slice was added above and below to the slice centred on the hilum at position “0”. Comparison of each of these datasets was performed using the Kruskal-Wallis test, which revealed that there was no significant difference in the mean %FF (*P* = 0.31). Post-hoc analysis demonstrated no significant pairwise differences for any comparisons between 1 and 11 slices. A number of significant pairwise differences were present between the 17 and 19 slice datasets, which included the most cranial and caudal slices, and all other slices. The Kruskal-Wallis test demonstrated a significant difference in the multiple comparisons of coefficient of variance (*P* < 0.001) (Fig. 5). Post hoc analysis revealed that significant differences were present in all pairwise comparisons except between the “3” and “5” slice datasets (*P* = 0.19).

## Discussion

While drawing regions of interest it became evident that the most cranial and caudal slices through the liver were prone to uncharacteristically high %FF values. This can be explained by the inclusion of the surrounding intra-abdominal and peri-hepatic fat within the most cranial and most caudal voxels by partial volume effect. The same finding was also noted by Kim et al. when evaluating different ROI measurements using MRS [9].

Our study demonstrated that left lobe %FF measured, using an ellipsoid ROI, was significantly different to the right lobe, with the %FF values for the left lobe being higher. A higher %FF in the left lobe has not been reported by previous studies, which have suggested that there is either no significant difference [20, 26] or that the %FF within the right lobe is higher than the left [9, 27, 28]. Reasons for this difference could relate to technical factors. Difficulty drawing a representative ellipse on some slices because of the small size of the left lobe might have introduced partial volume effects, or the inclusion of small vessels in the large right ROI could have contributed to a reduced %FF, although not all prior studies have excluded small vessels from their ROIs. Population differences between studies could also account for these findings, the current study is the first to assess multiple healthy volunteers from a range of different BMIs.

A significant difference was found when comparing the median %FF obtained using the freehand whole liver ROI and the elliptical right lobe ROI in both observers (*p* < 0.001). This finding might suggest that the simpler, quicker method of drawing an ellipse cannot replace the more laborious freehand drawn ROI. This result is supported by similar findings reported by Vu et al. and Hong et al. who suggest that sampling a single lobe using combinations of smaller ROIs is not sufficient. Both of these studies have suggested the use of ROI strategies that sample at minimum both lobes, and if possible each liver segment [20, 29].

When the difference between the mean right elliptical ROI and the reference freehand whole liver ROI values was calculated, it was found to be at most 2.79%. If a 3% margin of error is a clinically acceptable trade-off for a faster measurement in day-to-day use, then an elliptical ROI of the right lobe could still be used as a faster way to measure mean %FF. However, although not universally agreed, a commonly quoted %FF cut-off value for defining “hepatic steatosis” using MRI-PDFF is 5.6% [30]. Important decisions such as transplant donor acceptability may be made using this threshold [15]. In these cases a %FF value derived from an elliptical ROI could vary by up to 3% from the reference, a patient could only be acceptably classified as “steatotic” when their elliptical right lobe ROI was > 9%. This is likely to be too high to have practical uses.

Our study measured %FF on every slice that contained visible liver parenchyma. From this data set it was possible to establish whether the number of slices used to calculate the average %FF caused this to change significantly. A Kruskal-Wallis analysis demonstrated that when using a freehand drawn whole liver ROI there is no significant difference in the overall mean %FF if only one slice was sampled compared to sampling multiple slices. This suggests that one freehand whole liver ROI drawn on a single slice will provide a %FF that is representative of the entire liver.

On the other hand the variance of %FF values increased as more slices were included in the calculations. This trend was seen with both freehand whole liver ROI and elliptical ROI methods. This result is likely to be attributable to the heterogeneous distribution of fat within the liver e.g. due to anatomical variance of focal fatty infiltration. Therefore if a measure of the heterogeneity of liver fat distribution is of clinical interest, then an accurate measurement can only be obtained by using ROIs on a large number of slices. However as mentioned previously, extending the number of slices to include the most cranial and caudal sections may reduce the accuracy of whole liver %FF measurement.

The results showed “excellent” inter-observer agreement for both the freehand whole liver ROI and elliptical ROI protocols, with narrow limits of agreement, which suggests that both types of ROI yield reproducible results.

This study has some limitations relating to standardization of method compared to previous studies. These measurements were made using a vendor specific (IDEAL IQ) sequence on a wide bore 3 T MR machine and therefore may be specific to this arrangement, although a previously published study has demonstrated good reproducibility between different platforms when using IDEAL IQ [31]. In our study no attempt was made to exclude small hepatic vessels and bile ducts from the ROI. This methodology is similar to other published studies [20]. Although some studies using smaller ROIs that do not sample the whole liver parenchyma have avoided intrahepatic vessels, [17, 21, 32] it is not known whether or not this significantly affects accuracy.

## Conclusions

Our study is the first to assess %FF sampling the use of large, solitary, elliptical ROIs focused on one hepatic lobe as an alternative to using freehand drawn ROIs that enclose the whole liver. Our findings, consistent with prior studies demonstrate that the difference between %FF obtained when using these simplified methods is unlikely to be clinically acceptable. We would therefore recommend that freehand drawn ROIs encompassing the whole liver are used in preference.

Additionally, this study assesses the impact that varying the number of slices analysed has on the average %FF obtained when compared to whole liver %FF. Our findings suggest that there is no significant difference in the overall mean %FF if only one slice was sampled compared to sampling multiple slices. However, if insight into the variability of liver fat deposition is of interest, then multiple slices that contain visible liver parenchyma should be assessed. Although the data suggests that measurements from the most cranial and caudal slices show marked differences in %FF that are likely to be artefactual and inclusion of these slices may reduce accuracy.

Both the simplified elliptical ROI and freehand ROI methods demonstrated good inter-observer reliability and consistency at a wide range of BMI measurements.

### Conclusions in brief

- The use of large, solitary, elliptical ROIs focused on one hepatic lobe provides %FF measurements that are unlikely to be sufficiently accurate for use in clinical practice. Freehand whole-liver ROIs should be used in preference.
- When using freehand ROI measurements analysis of a single slice at the level of the hepatic hilum yields a %FF that is representative of the mean % FF of the whole liver.
- Both the simplified elliptical ROI and freehand ROI methods demonstrated good inter-observer reliability and consistency at a wide range of BMI measurements.

## Abbreviations

%FF: Percentage fat fraction
CV: Coefficient of variation
fh-ROI: Free-hand (whole liver) region of interest
HCC: Hepatocellular carcinoma
IDEAL IQ: Iteraterative decomposition of water and fat with echo asymmetry and least-squares estimation quantitation sequence
IQR: Inter quartile range
lt-ROI: Elliptical left lobe region of interest
MRI: Magnetic resonance imaging
MRS: Magnetic resonance spectroscopy
NAFLD: Non-alcoholic fatty liver disease
NASH: Non-alcoholic steatohepatitis
PDFF: Proton density fat fraction
ROI: Region of interest
rt-ROI: Elliptical right lobe region of interest
